# Prognostic factors in patients with septic shock in digestive surgery who have undergone direct hemoperfusion with polymyxin B-immobilized fibers: a retrospective observational study

**DOI:** 10.1186/s40560-015-0078-3

**Published:** 2015-03-13

**Authors:** Satoshi Matsukuma, Kazuhiko Sakamoto, Mitsuo Nishiyama, Takao Tamesa, Shigefumi Yoshino, Shoichi Hazama, Rumi Oshibuchi, Norimasa Matsuda, Satoshi Matsumoto, Hiroya Wakamatsu, Ryosuke Tsuruta, Mishiya Matsumoto, Masaaki Oka

**Affiliations:** Department of Digestive Surgery and Surgical Oncology, Yamaguchi University Graduate School of Medicine, Minami-Kogushi 1-1-1, Ube City, Yamaguchi Prefecture Japan; Intensive Care Unit, Yamaguchi University Hospital, Ube City, Yamaguchi Prefecture Japan; Advanced Medical Emergency and Critical Care Center, Yamaguchi University Hospital, Ube City, Yamaguchi Prefecture Japan

**Keywords:** Intra-abdominal infection, Hemodynamic dysfunction, Prognostic score

## Abstract

**Background:**

Direct hemoperfusion with polymyxin B-immobilized fiber (PMX-DHP) has been widely used for patients with septic shock around the world, but the prognostic factors have not been fully understood. We conducted a retrospective analysis to determine the prognostic factors in patients with septic shock who underwent PMX-DHP.

**Methods:**

Twenty-nine patients with septic shock who underwent PMX-DHP were included in the study. The patients were divided into groups based on survival (*n* = 23) and non-survival (*n* = 6) 28 days after PMX-DHP, and the clinical data for the two groups before and after PMX-DHP were compared.

**Results:**

In non-survivors, the vasopressor dependency index before PMX-DHP was significantly higher (*p* = 0.046), and the leukocyte count before PMX-DHP was significantly lower (*p* = 0.024) than in survivors. Furthermore, base excess after PMX-DHP was significantly lower in non-survivors (*p* = 0.007) than in survivors. The optimal cutoff points of the vasopressor dependency index, leukocyte count, and base excess identified by receiver operating characteristic curves were 0.499/mmHg, 1360/μL, and −6.4 mmol/L, respectively. And the score using these three cutoffs, termed the prognostic score, was related to the prognosis of septic shock patients who underwent PMX-DHP (area under the curve = 0.946).

**Conclusions:**

The prognostic score, using three parameters which are immediately and readily available in early phase after starting PMX-DHP, might be useful to predict the prognosis of these patients.

## Background

Sepsis is a systemic inflammatory response syndrome induced by infection [1]. Severe sepsis and septic shock affect 15 to 19 million people worldwide each year [2], mortality rate is declining as a result of advances in treatment, but is still 20% to 30% [3,4]. Intra-abdominal infection is a main focus of severe sepsis and septic shock [5], and a crucial problem in surgical critical care. The Surviving Sepsis Campaign emphasized the importance of source control, adequate antibiotics therapy, and hemodynamic support in the early phase of sepsis management [6], which is termed early goal directed therapy [7]. Antibiotic and surgical therapies for source control are essential, but these approaches cannot remove endotoxins and the endogenous mediators already released into blood.

Polymyxin B-immobilized fiber (PMX) is a commercially available column that is covalently bound to polymyxin B (Toraymyxin; Toray Industries, Tokyo, Japan). Its structure prevents the elution of polymyxin B and protects patients from nephrotoxicity and neurotoxicity [8]. PMX is believed to mainly adsorb endotoxin, but recent studies have reported that it also adsorbs anandamide, inducing hypotension, immunosuppression, and cytotoxicity [9], and reduces serum cytokine levels [10,11], monocyte messenger RNA expression [12], and the percentage of CD4^+^ CD25^+^ Forkhead box protein 3(Foxp3)^+^ T cells, termed regulatory T cells in the CD4^+^ T cell population [13].

It is known that direct hemoperfusion with PMX (PMX-DHP) has various effects on septic shock, and a few studies have shown that it has an ameliorating effect on prognosis [14]. Although a preliminary randomized controlled trial showed that PMX-DHP improved hemodynamic and respiratory dysfunction and reduced 28-day mortality in patients with severe sepsis and septic shock due to intra-abdominal infection [15], its improvement effect on prognosis remains controversial [16]. And two randomized controlled trials are ongoing in France (Effects of Hemoperfusion With a Polymyxin B Membrane in Peritonitis With Septic Shock (ABDO-MIX); ClinicalTrials.gov identifier: NCT01222663) and the United States (Safety and Efficacy of Polymyxin B Hemoperfusion (PMX) for Septic Shock (EUPHRATES); ClinicalTrials.gov identifier: NCT01046669). Before discussing the role of PMX-DHP in the management of patients with severe sepsis and septic shock, we should consider the forthcoming results of these ongoing trials.

It is unclear which patients will benefit from PMX-DHP and survive septic shock. If we could predict the prognosis of septic shock patients who undergo PMX-DHP, we would be able to identify the patient in danger of death and make risk stratification for future novel therapies. To determine the predictive factors of survival in the early phase after starting PMX-DHP and make clinical prediction rule for survival, we conducted a retrospective analysis of patients with septic shock who underwent PMX.

## Methods

### Patients

Twenty-nine consecutive patients with septic shock who underwent PMX-DHP between January 2006 and December 2013 in the Department of Digestive Surgery and Surgical Oncology at Yamaguchi University Hospital were enrolled in this retrospective study. This study was approved by the institutional review board of Yamaguchi University. The review board approved the waiving of the informed consent because this study consisted of a historical cohort.

All patients were followed up until discharge or death. The patients were divided into groups based on survival (*n* = 23) and non-survival (*n* = 6) 28 days after PMX-DHP, and the clinical data for the two groups before and after PMX-DHP were compared.

### Initial resuscitation

Administration of catecholamine was started to keep the patient’s mean arterial pressure (MAP) ≥65 mmHg despite adequate fluid resuscitation. We used noradrenaline as first-choice vasopressor and added vasopressin (0.03 U/min) if needed. Dobutamine infusion was added to vasopressor in patients with myocardial dysfunction [6]. Procedures for source control were conducted as soon as possible, if feasible. The presumptive therapies with broad spectrum antibiotics were started as soon as possible, and switched to definitive therapies with narrower spectrum agents after culture results and antimicrobial susceptibility data returned.

### PMX-DHP

PMX-DHP was performed for patients with hemodynamic instability in spite of administration of catecholamine through a double-lumen catheter inserted in the cervical vein or the femoral vein at a blood flow rate of 80–100 mL/min with 20 mg/h nafamostat mesilate, as the anticoagulant. Treatment was performed for longer than 2 h and up to 24 h because some reports have suggested that an extended duration of treatment may be beneficial [17]. All patients underwent one or two sessions of PMX-DHP.

### Clinical data

Clinical data were recorded, and the Acute Physiology and Chronic Health Evaluation (APACHE II) score [18], Sequential Organ Failure Assessment (SOFA) score [19], and disseminated intravascular coagulation score from the Japanese Association of Acute Medicine [20] was determined at the start of PMX-DHP.

The inotropic score [21,22] is the most commonly used index in critical care medicine for expressing hemodynamic dysfunction and is calculated as follows (all doses are expressed as μg/kg/min): (dopamine dose × 1) + (dobutamine dose × 1) + (adrenaline dose × 100) + (noradrenaline dose × 100) + (phenylephrine dose × 100). Because this score has different meaning for targeted arterial pressure, we adopted the vasopressor dependency index (VDI) [15], which was introduced in the Early Use of Polymyxin B Hemoperfusion in Abdominal Sepsis (EUPHAS) trial and is calculated as the ratio of the inotropic score to the MAP.

The uses of continuous renal replacement therapy, antithrombin, recombinant-soluble thrombomodulin, and intravenous immunoglobulin were recorded. Procedures for source control included drainage for abdominal abscess.

We recorded the MAP, VDI, improvement rate of the VDI, leukocyte count, data on arterial blood gases, improvement rate of the PaO_2_/FIO_2_ ratio, and lactate clearance rate 6 h after the start of PMX-DHP. Although duration of PMX-DHP differed from patients to patients, we chose 6 h after the start of PMX-DHP instead of completion of that to evaluate every patient at the same point in time and determine the prognostic factors in the early phase after starting PMX-DHP.

### Statistical analysis

Categorical variables are presented as numbers and were analyzed by using Fisher’s exact test. Continuous variables were compared by using the Mann–Whitney *U* test and data are presented as medians and ranges. We selected the predictive factors of 28-day survival, constructed receiver operating characteristic (ROC) curves of each factor and determined the area under the curve (AUC). The optimal cutoff point for balancing the sensitivity and specificity of each factor was identified as the point on the ROC curve closest to the upper left-hand corner. Subsequently, we allocated one point to each of the prognostic factor which was worse than the respective cutoff value, and calculated the total score of each patient, termed the prognostic score, by adding these points. The Kaplan-Meier method and log-rank test were performed for survival analysis of each score. We constructed ROC curve of the prognostic score for the prediction of 28-day survival and determined the AUC. A *p* value ≤0.05 was considered statistically significant. Data were analyzed with StatFlex version 6.0 (Artec, Osaka, Japan).

## Results

### Baseline patient characteristics

The demographic data before PMX-DHP are shown in Tables 1 and 2. Of the 29 patients, 23 patients were alive and 6 patients had died 28 days after PMX-DHP. The proportion of cases of intra-abdominal infection was greater in survivors than in non-survivors (*p* = 0.013).

**Table 1:** **Baseline patient characteristics**

	**Survivors** **(** ***n*** **= 23)**	**Non-survivors** **(** ***n*** **= 6)**	***P*** **values**
Age (years)	67.0 (27–95)	74.5 (58–83)	0.269
Gender (male/female)	14/9	4/2	0.592
Site of infection (number of patients)			
Intra-abdominal infection	21	3	0.013
Pneumonia	1	0	
unidentified source	1	1	
CRBSI	0	1	
Vibriovulnificus infection	0	1	
Procedures for source control (+/−)	20/3	4/2	0.269

Data are presented as medians (ranges).

*Abbreviations*: *CRBSI* catheter-related bloodstream infection.

**Table 2:** **Baseline patient characteristics**

	**Survivors** **(** ***n*** **= 23)**	**Non-survivors** **(** ***n*** **= 6)**	***P*** **values**
ICU admission to PMX-DHP start (min)	62 (15–2,317)	75 (20–1,170)	0.483
Number of PMX-DHP per patient (1/2)	19/4	4/2	0.363
Time per treatment (min)			
1^st^ session	325 (50–1,835)	820 (200–1,340)	0.161
2^nd^ session	1,080 (420–1,440)	1,372 (1,330–1,414)	NA
Total time of PMX-DHP (min)	360 (50–2,880)	1,330 (490–2,455)	0.091
CRRT (+/−)	14/9	5/1	0.302

Data are presented as medians (ranges).

*Abbreviations*: *ICU* intensive care unit, *PMX-DHP* direct hemoperfusion with polymyxin B-immobilized fiber, *NA* not available, *CRRT* continuous renal replacement therapy.

We did not conduct procedures for source control in five patients. There were two patients without identified source, a patient with pneumonia and a patient with systemic vibrio vulnificus infection. And a patient with abdominal abscess after hepatectomy did not undergo drainage because of marked bleeding tendency, and survived to discharge.

Of the 29 patients, 24 patients were treated with carbapenem or piperacillin/tazobactam agents as initial therapies. And vancomycin or daptomycin or linezolid were used concurrently if methicillin-resistant *Staphylococcus aureus* or *Enterococcus species* infection were possible pathogens.

There were no differences between the two groups in use of antithrombin, recombinant soluble thrombomodulin, and intravenous immunoglobulin (data not shown).

### Comparison of the clinical features and laboratory data before PMX-DHP between survivors and non-survivors

In non-survivors, the VDI before PMX-DHP was significantly higher (*p* = 0.046) and the leukocyte count before PMX-DHP was significantly lower (*p* = 0.024) than in survivors (Tables 3 and 4). We were not able to evaluate which population of leukocytes decreased in non-survivors because differential leukocyte counts were not consistently available. There were no other significant differences between the two groups.

**Table 3:** **Comparison of the clinical features before PMX-DHP between survivors and non-survivors**

	**Survivors** **(** ***n*** **= 23)**	**Non-survivors** **(** ***n*** **= 6)**	***P*** **values**
Respiratory rate (**/**min)	16 (10–25)	16.5 (12–30)	0.745
Bladder temperature (Celsius)	36.9 (34.2–38.8)	36.2 (34.4–37.9)	0.536
Heart rate (/min)	105 (67–135)	120 (80–130)	0.186
MAP (mmHg)	70 (50–117)	59 (40–82)	0.100
Inotropic score	14.1 (2.3–44.7)	45.5 (5.5–50.6)	0.095
VDI (**/**mmHg)	0.195 (0.025–0.645)	0.562 (0.080–0.802)	0.046
APACHE II score	15 (8–26)	22 (8–29)	0.177
SOFA score	7 (2–13)	8.5 (6–15)	0.099
DIC score	2 (0–8)	4.5 (2–6)	0.156

Data are presented as medians (ranges).

*Abbreviations*: *MAP* mean arterial pressure, *VDI* vasopressor dependency index, *APACHE* II acute physiology and chronic health evaluation, *SOFA* sequential organ failure assessment, *DIC* disseminated intravascular coagulation.

**Table 4:** **Comparison of the laboratory data before PMX-DHP between survivors and non-survivors**

	**Survivors** **(** ***n*** **= 23)**	**Non-survivors** **(** ***n*** **= 6)**	**P values**
Leukocytes (/μl)	6,420 (1,370–25,220)	1,110 (430–16,540)	0.024
Ht (%)	33.3 (23.8–44.9)	34.4 (19.2–45.5)	0.957
Platelets (×10^4^/μl)	14.3 (3.2–42.0)	8.5 (5.2–19.1)	0.053
Total bilirubin (mg/dl)	0.6 (0.4–8.2)	1.0 (0.8–2.0)	0.110
Creatinine (mg/dl)	0.98 (0.36–3.36)	1.18 (0.72–2.82)	0.132
d-dimer (μg/ ml)	11.2 (1.0–502.9)	7.8 (3.7–16.6)	0.448
C-reactive protein (mg/dl)	6.75 (0.26–27.38)	7.07 (0.34–13.05)	0.477
Arterial blood gases			
pH	7.354 (7.249–7.469)	7.325 (7.208–7.404)	0.258
Base excess (mmol/l)	−3.80 (−10.2–3.1)	−6.10 (−8.10–1.60)	0.170
P/F (mmHg)	229 (91–525)	195 (102–395)	0.360
Lactate (mmol/l)	3.3 (0.6–8.7)	4.8 (1.7–7.8)	0.389

Data are presented as medians (ranges).

*Abbreviation*: *P/F* PaO_2_/FlO_2_ ratio.

### Comparison of clinical features 6 h after the start of PMX-DHP between survivors and non-survivors

The pH and base excess (BE) of arterial blood of non-survivors were significantly lower than those of survivors (*p* = 0.018 and 0.007, respectively) (Table 5). The lactate level was significantly higher in non-survivors (*p* = 0.043). The VDI after PMX-DHP decreased significantly (*p* = 0.0036) compared with the VDI before PMX-DHP only in survivors (data not shown). There was no significant difference in leukocyte count between survivors and non-survivors. And it did not show significant changes between before- and after PMX-DHP in both groups (data not shown). There were no significant differences in the VDI, improvement rate of the VDI, improvement rate of the PaO_2_/FIO_2_ ratio, and lactate clearance rate.

**Table 5:** **Comparison of clinical features 6 h after the start of PMX-DHP between survivors and non-survivors**

	**Survivors** **(** ***n*** **= 23)**	**Non-survivors** **(** ***n*** **= 6)**	***P*** **values**
MAP (mmHg)	75 (45–101)	79 (42–98)	0.914
VDI (/mmHg)	0.106 (0.000–0.569)	0.256 (0.130–0.612)	0.076
Improvement rate of VDI (%)	39.3 (−116.9–100.0)	39.2 (−61.4–74.1)	0.957
Leukocytes (/μl)	5,700 (1,050–31,200)	3,800 (1,400–11,100)	0.200
Arterial blood gas			
pH	7.395 (7.305–7.458)	7.329 (7.130–7.388)	0.018
Base excess (mmol/l)	−1.7 (−6.7–5.5)	−7.2 (−12.9–0.2)	0.007
P/F (mmHg)	267 (108–483)	222 (50–379)	0.132
Improvement rate of P/F (%)	9.0 (−44.8–228.2)	59.2 (−76.5–169.8)	0.518
Lactate (mmol/l)	2.9 (0.8–8.8)	6.5 (1.3–9.9)	0.043
Lactate clearance (%)	10.0 (−83.3–61.9)	−6.6 (−65.0–23.5)	0.060

Data are presented as medians (ranges).

*Abbreviations*: *MAP* mean arterial pressure, *VDI* vasopressor dependency index, *P/F* PaO_2_/F1O_2_ ratio.

### Analysis of ROC curves

**Figure 1:**
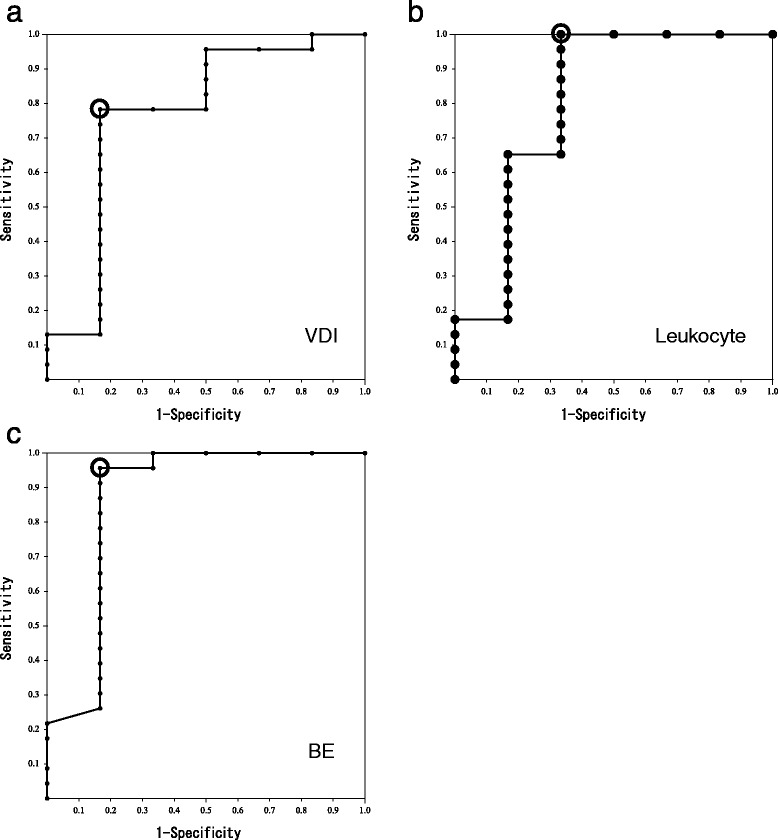
**Receiver operating characteristic (ROC) curves for the prediction of 28-day survival. (a)** ROC curve of the vasopressor dependency index (VDI). The optimal cutoff point is represented as an open circle. The area under the curve (95% confidence interval) and optimal cutoff points (sensitivity, specificity) of the vasopressor dependency index were 0.768 (0.514–1.000) and 0.499/mmHg (78.3%, 83.3%), respectively. **(b)** ROC curve of the leukocyte count. The optimal cutoff point is represented as an open circle. The area under the curve (95% confidence interval) and optimal cutoff points (sensitivity, specificity) of the leukocyte count were 0.804 (0.551–1.000) and 1360/μL (100%, 66.7%), respectively. **(c)** ROC curve of base excess (BE). The optimal cutoff point is represented as an open circle. The area under the curve (95% confidence interval) and optimal cutoff points (sensitivity, specificity) of base excess were 0.866 (0.647–1.000) and −6.4 mmol/L (95.7%, 83.3%), respectively.

We constructed ROC curves of the VDI (Figure 1a) and leukocyte count (Figure 1b) before PMX-DHP for the prediction of 28-day survival and determined the AUC. The AUC (95% CI) of the ROC curves of the VDI and leukocyte count were 0.768 (0.514–1.000) and 0.804 (0.551–1.000), respectively. The optimal cutoff points (sensitivity, specificity) of the VDI and leukocyte count were 0.499/mmHg (78.3%, 83.3%) and 1360/μL (100%, 66.7%), respectively.

The BE, pH, and lactate level after PMX-DHP were significantly correlated, and regression coefficients between BE and pH, pH and lactate level, and BE and lactate level were 0.8095, −0.5167, and −0.6373, respectively. To compare the prediction abilities of these factors, we constructed ROC curves. Because the AUC of the ROC curve of BE was greater than that of pH and lactate level (BE: 0.866 (95% CI 0.647–1.000), pH: 0.819 (95% CI 0.638–0.999), lactate level: 0.772 (95% CI 0.505–1.000)), we considered BE to be the superior prognostic factor. We judged the optimal cutoff point of BE after PMX-DHP to be −6.4 mmol/L, and the sensitivity and specificity at this point were 95.7% and 83.3%, respectively (Figure 1c).

### Prognostic score and 28-day survival

**Figure 2:**
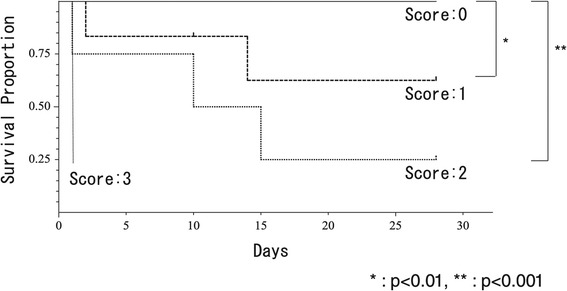
**Survival rate of each group.** We allocated one point to each of the three prognostic factors (vasopressor dependency index before PMX-DHP greater than 0.499/mmHg, leukocyte count before PMX-DHP less than 1360/μL, and base excess after PMX-DHP less than −6.4 mmol/L), and calculated the prognostic score by adding these points. All patients with a score of 0 (*n* = 18) were alive after 28 days, whereas the patient with a score of 3 (*n* = 1) died within 24 h. The survival rates of the patients with a score of 1 (*n* = 6) and a score of 2 (*n* = 4) were 67% and 25%, respectively. There were significant differences in survival rates between a score of 0 and a score of 1 (*p* = 0.0078) and between a score of 0 and a score of 2 (*p* < 0.0001). Abbreviation: PMX-DHP, direct hemoperfusion with polymyxin B-immobilized fiber.

Subsequently, we allocated one point to each of the three prognostic factors (VDI before PMX-DHP greater than 0.499/mmHg, leukocyte count before PMX-DHP less than 1360/μL, and BE after PMX-DHP less than −6.4 mmol/L), and calculated the prognostic score by adding these points. The 28-day survival rate for each score is shown in Figure 2. All patients with a score of 0 (*n* = 18) were alive after 28 days. The survival rates of patients with a score of 1 (*n* = 6) and a score of 2 (*n* = 4) were 67% and 25%, respectively. The patient with a score of 3 (*n* = 1) died within 24 h after the start of PMX-DHP. There were significant differences in survival rates between a score of 0 and a score of 1 (*p* = 0.0078) and between a score of 0 and a score of 2 (*p* < 0.0001). The number of patients with a score of 3 was too small for statistical analysis.

**Figure 3:**
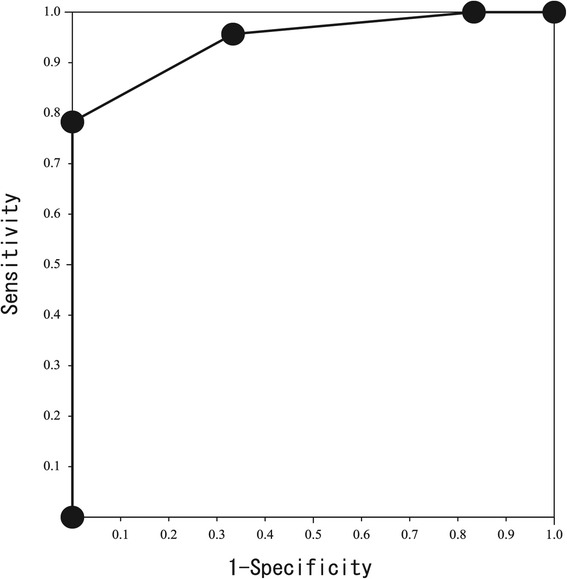
**Receiver operating characteristic curve of the prognostic score for the prediction of 28-day survival.** The area under the curve (95% confidence interval) was 0.946 (0.865–1.000) and greater than those of each three factors.

The ROC curve of the prognostic score for the prediction of 28-day survival is shown in Figure 3. The AUC (95% CI) was 0.946 (95% CI 0.865–1.000) and greater than those of each three factors.

## Discussion

The findings of this retrospective study suggest that the VDI and leukocyte count before PMX-DHP and BE after PMX-DHP may be prognostic factors, and the total score calculated with cutoff points for these factors could be related to the survival rate of patients with septic shock who undergo PMX-DHP. Some studies have reported prognostic factors in these patients, including APACHE II score [23,24], SOFA score [23], and HMGB-1 [25]. To the best of our knowledge, this is the first report suggesting that VDI and leukocyte count before PMX-DHP and BE after PMX-DHP are prognostic markers and the predictive score combined with these three factors.

The VDI, which was introduced in the EUPHAS trial [15], is a surrogate marker of hemodynamic status expressing a relationship between the dose of catecholamine and the response of MAP. There were no differences in MAP and inotropic score in this study, whereas the VDI in non-survivors was significantly higher than that in survivors. This result suggests that the VDI is a more sensitive marker of hemodynamic in patients with septic shock than the inotropic score. Kobayashi et al. reported that early improvement of VDI after PMX-DHP was a prognostic factor and a significant decrease in the VDI after PMX-DHP was seen only in survivors [26]. Because it is impossible to judge whether decreasing of the VDI in each patient is significant, we need to set a cutoff point or evaluate the improvement rate of the VDI for prediction of prognosis. The AUC of the ROC curve of the VDI after PMX-DHP in our study for the prediction of 28-day survival was 0.739 (95% CI 0.544–0.934) and inferior to that before PMX-DHP. However, the cutoff point of the VDI before PMX-DHP determined in our study was also not sufficient for prognostic prediction; these results indicate that the VDI may be just one aspect of the severity of sepsis.

There were no significant differences in VDI and improvement rate of the VDI between the two groups 6 h after the start of PMX-DHP in this study. We think two main reasons for this. First, sample size was too small to detect significance because VDI in non-survivors after PMX-DHP tended to be higher than that in survivors (Table 5). Second, microcirculatory dysfunction could not be improved only by hemodynamic stabilization.

A decreased peripheral blood leukocyte count in the early phase of sepsis has been explained as the migration of leukocytes to the focus of infection or some organs induced by several cytokines, and it is an important feature for the diagnosis of sepsis [27]. Leukopenia has been reported as a prognostic factor of sepsis in some studies [28], and our finding suggests that it may also be important in patients with septic shock who have undergone PMX-DHP. Unfortunately, the differential leukocyte count was not assessed in almost half of our patients, so it is unknown which population is especially important for prognosis. Because leukopenia in patients with sepsis means that hematopoietic function cannot respond to inflammation, immunodeficiency could develop in these patients.

Acidemia, decreased BE, and hyperlactemia are consequences of tissue hypoperfusion and imbalance of oxygen delivery and consumption. Of these three factors, lactate has been focused on in the past. Lactate-guided therapy significantly reduces hospital mortality [29], and the Surviving Sepsis Campaign guideline recommends normalizing the lactate level as rapidly as possible [6]. On the other hand, Couto-Alves et al. have reported a scoring system using BE and platelet count at presentation for prognostic prediction in pediatric meningococcal sepsis [30]. Although the lactate level was also significantly higher among non-survivors in that study, BE was used to build a score because of its better sensitivity. The reason why BE was more sensitive than lactate is unknown, but this discrepancy could reflect increased fixed acids by impaired excretion that resulted from renal dysfunction or hypercatabolism caused by inflammation.

In this study, the proportion of septic patients with intra-abdominal infection was significantly higher in the survivors. Although there was no difference between survivors and non-survivors regarding whether procedure was performed, the fact that surgical procedures were more available for intra-abdominal infection compared with other infectious sources may improve survival. In addition, PMX-DHP may be more useful for intra-abdominal sepsis, as reflected in many studies of PMX-DHP designed for intra-abdominal sepsis alone.

We acknowledge that this study has some limitations. First, a small number of patients with septic shock attributable to various causes were analyzed, and we could not perform multivariate analysis because of the limited number of patients. For this reason, these findings cannot be generalized to the broader clinical situation based on this study alone. Second, the effectiveness of PMX-DHP itself is uncertain because this study was retrospective. Third, the predictive score, which was calculated by adding one point for each factor, is insufficient because the weighting of each factor was not considered. We need to identify prognostic factors in well-designed, prospective, randomized, controlled trials with a large number of patients.

## Conclusions

In conclusion, the VDI and leukocyte count at the start of PMX-DHP and BE 6 h after the start of PMX-DHP are related to prognosis of patients with septic shock who undergo PMX-DHP. The prognostic score using the respective cutoff values of these three factors may be useful to predict the prognosis of these patients.

## References

[1] Levy MM, Fink MP, Marshall JC, Abraham E, Angus D, Cook D (2003). 2001 SCCM/ESICM/ACCP/ATS/SIS International Sepsis Definitions Conference. Crit Care Med.

[2] Adhikari NK, Fowler RA, Bhagwanjee S, Rubenfeld GD (2010). Critical care and the global burden of critical illness in adults. Lancet.

[3] Angus DC, Linde-Zwirble WT, Lidicker J, Clermont G, Carcillo J, Pinsky MR (2001). Epidemiology of severe sepsis in the United States: analysis of incidence, outcome, and associated costs of care. Crit Care Med.

[4] Kumar G, Kumar N, Taneja A, Kaleekal T, Tarima S, McGinley E (2011). Nationwide trends of severe sepsis in the 21st century (2000–2007). Chest.

[5] Lagu T, Rothberg MB, Shieh MS, Pekow PS, Steingrub JS, Lindenauer PK (2012). Hospitalizations, costs, and outcomes of severe sepsis in the United States 2003 to 2007. Crit Care Med.

[6] Dellinger RP, Levy MM, Rhodes A, Annane D, Gerlach H, Opal SM (2013). Surviving Sepsis Campaign: international guidelines for management of severe sepsis and septic shock, 2012. Intensive Care Med.

[7] Rivers E, Nguyen B, Havstad S, Ressler J, Muzzin A, Knoblich B (2001). Early goal-directed therapy in the treatment of severe sepsis and septic shock. N Engl J Med.

[8] Ronco C (2014). Endotoxin removal: history of a mission. Blood Purif.

[9] Wang Y, Liu Y, Sarker KP, Nakashima M, Serizawa T, Kishida A (2000). Polymyxin B binds to anandamide and inhibits its cytotoxic effect. FEBS Lett.

[10] Tani T, Hanasawa K, Kodama M, Imaizumi H, Yonekawa M, Saito M (2001). Correlation between plasma endotoxin, plasma cytokines, and plasminogen activator inhibitor-1 activities in septic patients. World J Surg.

[11] Nakamura T, Ebihara I, Shimada N, Koide H (1998). Changes in plasma erythropoietin and interleukin-6 concentrations in patients with septic shock after hemoperfusion with polymyxin B-immobilized fiber. Intensive Care Med.

[12] Nakamura T, Ebihara I, Shimada N, Shoji H, Koide H (1998). Modulation of plasma metalloproteinase-9 concentrations and peripheral blood monocyte mRNA levels in patients with septic shock: effect of fiber-immobilized polymyxin B treatment. Am J Med Sci.

[13] Ono S, Kimura A, Hiraki S, Takahata R, Tsujimoto H, Kinoshita M (2013). Removal of increased circulating CD4 + CD25 + Foxp3+ regulatory T cells in patients with septic shock using hemoperfusion with polymyxin B-immobilized fibers. Surgery.

[14] Zhou F, Peng Z, Murugan R, Kellum JA (2013). Blood purification and mortality in sepsis: a meta-analysis of randomized trials. Crit Care Med.

[15] Cruz DN, Antonelli M, Fumagalli R, Foltran F, Brienza N, Donati A (2009). Early use of polymyxin B hemoperfusion in abdominal septic shock: the EUPHAS randomized controlled trial. JAMA.

[16] Iwagami M, Yasunaga H, Doi K, Horiguchi H, Fushimi K, Matsubara T (2014). Postoperative polymyxin B hemoperfusion and mortality in patients with abdominal septic shock: a propensity-matched analysis. Crit Care Med.

[17] Mitaka C, Tsuchida N, Kawada K, Nakajima Y, Imai T, Sasaki S (2009). A longer duration of polymyxin B-immobilized fiber column hemoperfusion improves pulmonary oxygenation in patients with septic shock. Shock.

[18] Knaus WA, Draper EA, Wagner DP, Zimmerman JE (1985). APACHE II: a severity of disease classification system. Crit Care Med.

[19] Vincent JL, Moreno R, Takala J, Willatts S, De Mendonca A, Bruining H (1996). The SOFA (Sepsis-related Organ Failure Assessment) score to describe organ dysfunction/failure. On behalf of the Working Group on Sepsis-Related Problems of the European Society of Intensive Care Medicine. Intensive Care Med.

[20] Gando S, Iba T, Eguchi Y, Ohtomo Y, Okamoto K, Koseki K (2006). A multicenter, prospective validation of disseminated intravascular coagulation diagnostic criteria for critically ill patients: comparing current criteria. Crit Care Med.

[21] Shore S, Nelson DP, Pearl JM, Manning PB, Wong H, Shanley TP (2001). Usefulness of corticosteroid therapy in decreasing epinephrine requirements in critically ill infants with congenital heart disease. Am J Cardiol.

[22] Wernovsky G, Wypij D, Jonas RA, Mayer JE, Hanley FL, Hickey PR (1995). Postoperative course and hemodynamic profile after the arterial switch operation in neonates and infants. A comparison of low-flow cardiopulmonary bypass and circulatory arrest. Circulation.

[23] Komatsu S, Shimomatsuya T, Nakajima M, Ono S, Maruhashi K (2006). Severity scoring systems for prognosis and efficacy of polymyxin B-immobilized fiber treatment for colonic perforation. Surg Today.

[24] Sugimoto K, Sato K, Maekawa H, Sakurada M, Orita H, Ito T (2013). Analysis of the efficacy of direct hemoperfusion with polymyxin B-immobilized fiber (PMX-DHP) according to the prognostic factors in patients with colorectal perforation. Surg Today.

[25] Ueno T, Ikeda T, Ikeda K, Taniuchi H, Suda S, Yeung MY (2011). HMGB-1 as a useful prognostic biomarker in sepsis-induced organ failure in patients undergoing PMX-DHP. J Surg Res.

[26] Kobayashi A, Iwasaki Y, Kimura Y, Kawagoe Y, Ujike Y (2010). Early recovery in hemodynamics after direct hemoperfusion with polymyxin B-immobilized fibers may predict mortality rate in patients with septic shock. J Anesth.

[27] Bone RC, Balk RA, Cerra FB, Dellinger RP, Fein AM, Knaus WA (1992). Definitions for sepsis and organ failure and guidelines for the use of innovative therapies in sepsis. The ACCP/SCCM Consensus Conference Committee. Am Coll Chest Physicians/Soc Crit Care Med Chest.

[28] Wester AL, Dunlop O, Melby KK, Dahle UR, Wyller TB (2013). Age-related differences in symptoms, diagnosis and prognosis of bacteremia. BMC Infect Dis.

[29] Jansen TC, van Bommel J, Schoonderbeek FJ, Sleeswijk Visser SJ, van der Klooster JM, Lima AP (2010). Early lactate-guided therapy in intensive care unit patients: a multicenter, open-label, randomized controlled trial. Am J Respir Crit Care Med.

[30] Couto-Alves A, Wright VJ, Perumal K, Binder A, Carrol ED, Emonts M (2013). A new scoring system derived from base excess and platelet count at presentation predicts mortality in paediatric meningococcal sepsis. Crit Care.

